# Mortality trends in U.S. adults with septic shock, 2005-2011: a serial cross-sectional analysis of nationally-representative data

**DOI:** 10.1186/s12879-016-1620-1

**Published:** 2016-06-14

**Authors:** Tadahiro Goto, Kazuki Yoshida, Yusuke Tsugawa, Michael R. Filbin, Carlos A. Camargo, Kohei Hasegawa

**Affiliations:** Department of Emergency Medicine, University of Fukui Hospital, Fukui, Japan; Department of Epidemiology, Harvard T. H. Chan School of Public Health, Boston, MA USA; Harvard Interfaculty Initiative in Health Policy, Harvard University, Boston, MA USA; Department of Emergency Medicine, Massachusetts General Hospital, Harvard Medical School, Boston, MA USA

**Keywords:** Septic shock, Mortality trends, Vasopressor, Pneumonia, Urinary tract infection, Intra abdominal infection

## Abstract

**Background:**

We aimed to investigate mortality trends in hospitalized patients with septic shock in the US. To achieve this objective, we tested hypothesis that mortality decreased in patients identified by the code of septic shock while mortality did not change in those with septic shock identified by vasopressor use.

**Methods:**

We conducted a serial cross-sectional analysis using Nationwide Inpatient Sample database from 2005 through 2011. First, we identified all adult patients aged ≥18 years hospitalized for septic shock by the following criteria: 1) primary *ICD-9* diagnosis of infection plus procedure code for vasopressor use, 2) primary *ICD-9* diagnosis of infection plus septic shock in non-primary field, and 3) primary *ICD-9* diagnosis of septic shock. Second, we stratified all identified patients by record of vasopressor use. The outcome of interest was year-to-year changes in the in-hospital all-cause mortality.

**Results:**

From 2005 to 2011, we identified 109,812 weighted hospitalizations with septic shock. Overall, there was a significant downward trend in in-hospital mortality (from 46 % in 2005 to 42 % in 2011; *P*_*trend*_ = 0.003); the adjusted mortality also decreased significantly (OR for comparison of 2005 with 2011, 0.98; 95 % CI, 0.96–1.00; *P* < 0.001). In stratified analysis, the mortality trend was not significant in the subgroup with vasopressor use (from 42 % in 2005 to 40 % in 2011; *P*_*trend*_ =0.57); similarly, the adjusted mortality did not change significantly (OR, 1.01; 95 % CI, 0.97–1.05; *P* =0.62). By contrast, there was a downward trend in mortality in the subgroup without vasopressor use (from 47 % in 2005 to 43 % in 2011; *P*_*trend*_ =0.002); likewise, the adjusted mortality decreased significantly (OR, 0.97; 95 % CI, 0.95–0.99; *P* =0.002)

**Conclusions:**

From 2005 to 2011, we found a modest decrease in in-hospital mortality among patients identified with septic shock. However, in the subgroup with vasopressor use, we found no significant change in mortality. Our data challenge the conventional wisdom that mortality in this population has improved during the last decade.

**Electronic supplementary material:**

The online version of this article (doi:10.1186/s12879-016-1620-1) contains supplementary material, which is available to authorized users.

## Background

Sepsis is a major public health problem in the US, with the annual national incidence of severe sepsis and septic shock reaching 3 million in 2009 [1]. Due to the rapid and steady increase in the number of these patients with associated healthcare resource use, the total direct costs for hospitalizations have increased markedly, up from $15.4 billion in 2003 to $24.3 billion in 2007 [2].

Despite decades of intense research and major technological advances, mortality for septic shock remains high, ranging from 22 to 50 % [3–5]. In the US, claim-based datasets have primarily been used to estimate nationwide mortality trends for septic patients and shown a decrease in the estimated mortality [1, 2, 6]. For example, a study reported that the mortality declined from approximately 40 % in 1998 to 30 % in 2009 [7]. However, the decreased mortality may have been overestimated in these studies [1, 2, 6, 7], owing to an artifact of changes in diagnostic coding [8–10]. New ICD-9 codes for sepsis syndromes (sepsis, severe sepsis, septic shock) were introduced in 2002 and 2003. Additionally, increased clinical vigilance and subsequent coding as a result of awareness campaigns might result in identification of patients with less severe illness [8, 11]. Given that variation in the mortality of septic shock depends on the methods used for database abstraction [1, 2], the actual mortality trend among patients with septic shock remains largely unclear.

In this context, we investigated mortality trends in US patients with septic shock by using data from the 2005 to 2011 Nationwide Inpatient Sample. We used a methodological approach that is less sensitive to ambiguous coding practices and one that identifies a septic population with undeniable hemodynamic consequences, (i.e., those with documented vasopressor requirement) [9]. We hypothesized that the mortality trend in septic shock defined by vasopressor use would be unchanged, while the mortality trend in patients identified based on septic shock ICD-9 diagnosis code alone would decrease.

## Methods

### Design and settings

This is a serial cross-sectional analysis using the data from the 2005 to 2011 releases of the Nationwide Inpatient Sample (NIS). The NIS is the largest all-payer US inpatient care database, with approximately 8 million hospitalizations added each year [12]. Developed as part of the Healthcare Cost and Utilization Project by the Agency for Healthcare Research and Quality (AHRQ), the NIS contains a 20 % stratified sample of all short-term, non-federal, non-rehabilitation hospitals. Stratification and weighting variables enable the calculation of national estimates and temporal trends, accounting for the complex sampling design and expanded sampling framework over time. Information on the location of disposition (e.g., a hospital ward or an intensive care unit) is not included in the NIS. Up to 25 discharge diagnoses (increased from 15 codes in 2009) are coded with the use of the *International Classification of Diseases, 9th Revision, Clinical Modification* (*ICD-9-CM*), with the first-listed diagnosis regarded as the primary reason for hospitalization. This study was approved by the institutional review boards of Massachusetts General Hospital with an informed consent waiver.

### Patients

First, on the basis of the general approach of Kumar et al. [6], we identified all adult patients aged ≥18 years hospitalized for septic shock by using the following criteria: 1) principal *ICD-9-CM* diagnosis of infection associated with major causes of sepsis (described below) plus recorded vasopressor use (code, 00.17) in any procedural field, 2) principal *ICD-9-CM* diagnosis of infection associated with major causes of sepsis plus diagnosis of septic shock (code, 785.52) in non-primary diagnosis field (not principal diagnosis) regardless of vasopressor use, or 3) principal diagnosis of septic shock (code, 785.52) regardless of vasopressor use. To avoid the misclassification and complexity in mortality, we focused on definite, simple, “septic shock” patients.

Second, we stratified all identified patients by the use of vasopressor: 1) patients with a recorded use of vasopressor (the subgroup with vasopressor use), and 2) those without (the subgroup without vasopressor use). Defining septic shock as *ICD-9-CM* code for infection listed as a principal diagnosis paired with the use of a vasopressor has been established [3, 6, 11, 13]. Because the proportion of patients with the primary diagnosis of septic shock accounted for less than 1 % of septic shock in the current study, we did not stratified by the definitions. To minimize the effect of ambiguous definitions (e.g., larger list of ICD-9 codes that might denote suspected infection), we focused on the four major causes of sepsis (see Additional file 1: Table S1) [9, 14–19]: pneumonia (codes, 481, 482, 483, 485, 486), urinary tract infection (codes, 590, 595.0, 595.2–4, 595.89, 595.9, 597, 598.00–01, 599.0), abdominal infections (codes, 008.45, 009, 540–542, 543.9, 562.01, 562.03, 562.11, 562.13, 567, 569.5, 569.61, 569.71, 569.83, 572, 574–576, 614, 616), and bacteremia (code, 790.7) [1, 6, 7, 9, 20].

### Covariates

The NIS contains information on patient characteristics, including demographics (age, sex, and race/ethnicity), primary insurance type, quartiles for estimated median household income, and patient comorbidities. Primary insurance types were categorized into Medicare, Medicaid, private, self-pay, and others. To adjust for potential confounding by patient-mix, 29 Elixhauser comorbidity measures were derived based on the *ICD-9-CM* codes using the AHRQ Comorbidity Software [21]. This risk adjustment tool has been validated extensively [22]. As the NIS does not contain unique patient identifiers, the unit of analysis was hospital discharge-level.

Hospital characteristics included geographic region, urban-rural status, teaching status, and hospital control and ownership. Geographic regions (North, East, South, Midwest, and West) were defined according to Census Bureau boundaries. Urban-rural status for the patient residence was defined based on National Center for Health Statistics [23].

### Outcome measure

The outcome of interest was year-to-year changes in the in-hospital all-cause mortality. In-hospital mortality was defined as the number of deaths divided by the total number of hospitalizations for septic shock.

### Statistical analyses

The frequency of hospitalizations for septic shock was estimated by weighting the patient-level discharge data in the NIS files using the weights provided. To examine the mortality trends in the patients with septic shock, we fit two analytical models. First, we fit an unadjusted model that included only the calendar year as the independent variable. Second, we fit a multivariable logistic regression model adjusting for both patient-level variables (age, sex, race/ethnicity, primary payer, household income, and 29 Elixhauser comorbidity measures) and hospital-level characteristics (region, hospital control and ownership, urban and rural distinction, and hospital teaching status). To address the possibility that adoption of coding practices over the study period led to an artifact of declining mortality, we repeated the above analysis stratified by record of vasopressor use. All analyses were performed with SAS-callable SUDAAN statistical software, version 11.0.0 (Research Triangle Institute, Research Triangle Park, NC, USA). Two-sided *P* <0.05 was considered statistically significant.

## Results

From 2005 to 2011, overall, we identified 22,260 hospitalizations for septic shock in the US, corresponding to 109,812 weighted hospitalizations. Of these, the subgroup with vasopressor use comprised a weighted estimate of 19,108 (17 %) patients and the subgroup without vasopressor use comprised a weighted estimate of 90,705 (83 %) patients. Table 1 shows overall patient and hospital characteristics over the study period. Overall, patients identified as septic shock were more likely to be aged 60 to 69 years (*P*_*trend*_ < 0.001) and to have comorbidities, such as diabetes, liver disease, renal failure, and solid tumors (all *P*_*trend*_ < 0.01). These trends were also observed both in the subgroup with vasopressor use (Additional file 1: Table S2) and the subgroup without vasopressor use (Additional file 1: Table S3).

**Table 1:** Patient and hospital characteristics in the overall patients hospitalized for septic shock in U.S., 2005–2011

Variables^a^	2005	2006	2007	2008	2009	2010	2011	P_trend_
Patient characteristics								
Unweighted sample, *n*								
Overall^b^	2827	2783	2996	3381	3438	3442	3393	
Subgroup with vasopressor use	484	523	545	518	559	645	607	
Subgroup without vasopressor use	2343	2260	2451	2863	2879	2797	2786	
Weighted sample, *n*								
Overall	13946	13628	14861	16563	17239	17292	16283	
Subgroup with vasopressor use	2392	2566	2664	2494	2789	3293	2910	
Subgroup without vasopressor use	11554	11062	12198	14069	14450	13999	13373	
Age, y								
18–29	2 (1–2)	1 (1–2)	2 (2–3)	2 (2–3)	2 (1–2)	2 (1–3)	2 (1–2)	0.46
30–39	3 (2–4)	3 (3–4)	3 (2–4)	3 (2–3)	3 (2–4)	3 (2–4)	3 (2–3)	0.26
40–49	7 (6–9)	8 (7–9)	9 (8–10)	8 (7–9)	7 (6–8)	8 (7–9)	7 (6–8)	0.26
50–59	15 (13–17)	16 (14–18)	14 (13–16)	16 (14–17)	17 (15–18)	15 (13–16)	16 (14–18)	0.58
60–69	19 (17–21)	19 (17–21)	20 (18–22)	21 (19–23)	21 (20–23)	22 (20–24)	24 (22–26)	<0.001
70–79	25 (23–28)	24 (22–27)	25 (22–27)	24 (22–26)	24 (22–26)	24 (22–27)	23 (20–25)	0.02
80–89	23 (20–26)	22 (20–25)	21 (19–24)	22 (19–24)	22 (20–24)	21 (19–23)	21 (19–23)	0.03
≥ 90	5 (4–6)	5 (4–6)	5 (4–6)	5 (4–6)	5 (4–6)	5 (4–6)	5 (4–5)	0.70
Male sex	49 (49–49)	49 (49–49)	50 (50–50)	50 (50–50)	50 (50–50)	50 (50–50)	50 (50–50)	0.09
Race/ethnicity								
Non-Hispanic white	61 (54–69)	54 (48–60)	54 (48–60)	57 (52–63)	63 (57–69)	64 (58–70)	65 (59–71)	<0.001
Non-Hispanic black	7 (6–9)	10 (8–12)	9 (6–12)	9 (7–11)	8 (7–10)	12 (9–12)	11 (9–13)	0.001
Hispanic	7 (5–9)	10 (8–13)	9 (6–11)	8 (6–10)	9 (7–11)	10 (8–12)	10 (8–13)	0.09
Asian/native/other	4 (3–5)	5 (4–6)	6 (5–7)	6 (5–7)	6 (5–8)	6 (4–7)	6 (5–7)	0.10
Unknown	20 (16–24)	21 (17–26)	22 (18–26)	20 (15–24)	14 (9–18)	9 (6–11)	8 (5–11)	<0.001
Primary health insurance								
Medicare	66 (60–72)	65 (60–71)	65 (59–70)	64 (59–69)	65 (60–69)	64 (59–69)	64 (60–69)	0.34
Medicaid	10 (08–12)	10 (08–12)	09 (07–11)	11 (09–12)	10 (08–11)	11 (09–13)	11 (09–12)	0.39
Private	19 (17–21)	18 (16–20)	20 (18–23)	20 (17–22)	20 (17–22)	18 (16–21)	19 (17–21)	1.00
Self-pay	4 (3–5)	4 (3–5)	3 (3–4)	3 (3–4)	4 (3–5)	4 (3–5)	3 (2–4)	0.63
Other	2 (1–2)	3 (2–4)	2 (2–3)	2 (1–3)	2 (2–3)	3 (2–3)	3 (2–4)	0.14
Estimated median household income								
0–25 percentile	27 (23–30)	28 (23–32)	29 (25–34)	28 (25–32)	28 (25–32)	30 (26–34)	29 (25–32)	0.23
26–50 percentile	26 (23–30)	25 (22–28)	25 (22–28)	26 (23–28)	25 (22–28)	25 (22–27)	24 (21–27)	0.27
51–75 percentile	24 (21–27)	24 (21–27)	23 (21–26)	22 (19–25)	23 (21–26)	24 (21–26)	27 (24–30)	0.11
76–100 percentile	23 (19–28)	23 (19–27)	22 (19–25)	24 (20–28)	23 (20–27)	22 (19–25)	20 (17–23)	0.23
Selected comorbidities								
Congestive heart failure	30 (28–33)	31 (29–33)	31 (29–33)	26 (24–28)	28 (26–30)	28 (26–29)	31 (29–33)	0.11
Pulmonary circulation disorders	3 (2–3)	3 (2–3)	5 (4–6)	6 (5–7)	7 (6–8)	7 (6–8)	8 (7–9)	<0.001
Diabetes, uncomplicated	15 (13–17)	16 (15–18)	18 (16–19)	19 (17–20)	19 (18–21)	18 (16–19)	21 (19–22)	<0.001
Liver disease	5 (4–6)	5 (4–6)	5 (4–6)	5 (4–6)	6 (5–7)	6 (5–7)	7 (6–8)	0.002
Renal failure	14 (13–16)	20 (19–22)	21 (20–23)	21 (19–23)	23 (21–24)	22 (20–23)	24 (22–26)	<0.001
Solid tumor without metastasis	4 (4–5)	3 (3–4)	4 (4–5)	4 (3–5)	5 (4–5)	5 (4–5)	5 (5–6)	0.006
Hospital characteristics								
Region								
Northeast	29 (23–35)	26 (22–31)	24 (20–27)	22 (18–25)	20 (17–24)	22 (18–26)	20 (16–24)	0.03
Midwest	21 (18–25)	21 (17–25)	21 (17–25)	25 (21–28)	25 (21–30)	22 (18–27)	23 (19–28)	0.37
South	30 (26–34)	31 (27–34)	31 (27–35)	32 (28–36)	32 (29–35)	37 (32–41)	37 (33–42)	0.02
West	19 (16–22)	22 (19–26)	24 (20–28)	21 (18–25)	23 (19–26)	19 (16–22)	19 (16–22)	0.53
Location/teaching status								
Rural	11 (9–13)	8 (7–10)	10 (8–13)	8 (7–9)	9 (7–11)	9 (7–12)	9 (7–12)	0.45
Urban nonteaching	47 (41–53)	42 (37–46)	43 (38–47)	43 (38–47)	45 (40–49)	43 (39–47)	43 (38–47)	0.47
Urban teaching	41 (35–47)	50 (44–56)	47 (41–53)	49 (44–55)	46 (41–52)	48 (41–54)	48 (42–54)	0.57
Hospital control/ownership								
Government	27 (23–30)	28 (23–32)	29 (25–34)	28 (25–32)	28 (25–32)	30 (26–34)	29 (25–32)	0.97
Private, non-profit	26 (23–30)	25 (22–28)	25 (22–28)	26 (23–28)	25 (22–28)	25 (22–27)	24 (21–27)	0.18
Private, invest-own	24 (21–27)	24 (21–27)	23 (21–26)	22 (19–25)	23 (21–26)	24 (21–26)	27 (24–30)	0.02
Others	23 (19–28)	23 (19–27)	22 (19–25)	24 (20–28)	23 (20–27)	22 (19–25)	20 (17–23)	0.53

Data are expressed as % (95 % CI) unless otherwise indicated

^a^Percentages may not equal 100 due to rounding

^b^The number of overall patients may not equal the sum of the vasopressor group and non–vasopressor group due to rounding

### Mortality trends in patients with septic shock, 2005-2011

**Fig. 1:**
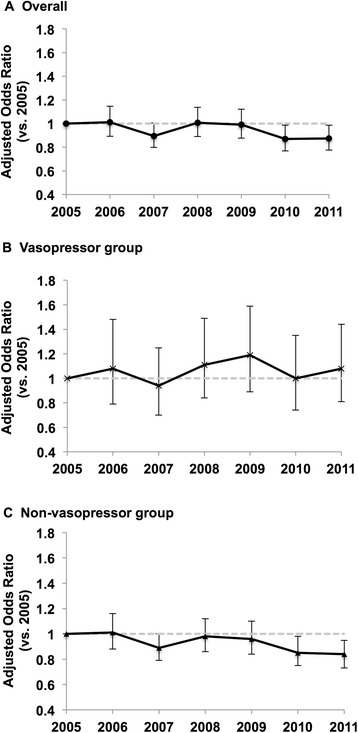
Trends in Adjusted Mortality for Patients with Septic Shock, 2005–2011. **a** In patients with septic shock, adjusted mortality significantly decreased (odds ratio for comparison of 2005 with 2011, 0.98; 95 % CI, 0.96–1.00). **b** In septic shock patients with recorded vasopressor use, adjusted mortality did not change significantly (odds ratio for comparison of 2005 with 2011, 1.01; 95 % CI, 0.97-1.05). **c** In septic shock patients without recorded vasopressor use, adjusted mortality significantly decreased (odds ratio for comparison of 2005 with 2011, 0.97; 95 % CI, 0.95–0.99)

Among all patients identified with septic shock, in-hospital mortality decreased from 46 % in 2005 to 42 % in 2011 (*P*_*trend*_ = 0.003; Additional file 1: Table S4). The multivariable-adjusted mortality also decreased significantly from 2005 to 2011 (odds ratio [OR] for comparison of 2005 with 2011, 0.98; 95 % CI, 0.96–1.00; *P* < 0.001; Fig. 1 and Additional file 1: Table S5).

In the stratified analysis, the mortality trend was not significant in the subgroup with vasopressor use (from 42 % in 2005 to 40 % in 2011; *P*_*trend*_ =0.57; Additional file 1: Table S4); similarly, the adjusted mortality did not change significantly (OR for comparison of 2005 with 2011, 1.01; 95 % CI, 0.97–1.05; *P* =0.62; Fig. 1 and Additional file 1: Table S5). By contrast, there was a downward trend in mortality in the subgroup without vasopressor use (from 47 % in 2005 to 43 % in 2011; *P*_*trend*_ =0.002); likewise, the adjusted mortality decreased significantly (OR for comparison of 2005 with 2011, 0.97; 95 % CI, 0.95–0.99; *P* =0.002).

## Discussion

In this serial cross-sectional study, using nationally-representative US samples from 2005 to 2011, we found an approximate 10 % relative decrease in in-hospital mortality among all adult patients identified with septic shock. However, in the subgroup with recorded vasopressor use, we found no significant change in mortality. In both septic shock subgroups, in-hospital mortality was unacceptably high, with case fatality rates of greater than 40 %.

Mortality trends in septic shock have important implications for clinical practice, research, and policy development. In the US, several studies using administrative data have indicated that the mortality among patients with septic shock decreased by approximately 6 to 10 % over the past decade. These reported declines in mortality might be driven by improvements in treatment strategies for patients with severe sepsis and septic shock [1, 2, 6]. Alternatively, mortality trends are subject to changes in the study population [1, 2]. Indeed, a previous study reporting a decrease in the mortality of septic shock from 1998 to 2009 found a concurrent 6-fold increase in the incidence of hospitalizations for septic shock during the same period, with an large step-increase around 2003 —the year that the *ICD-9-CM* specific code for septic shock (785.52) was created [7]. Similarly, another study reported a steady increase in hospitalization rates for sepsis that was paralleled by a stable or decreasing hospitalization rate for the infections that are the major causes of sepsis (e.g., pneumonia) [9]. Furthermore, our previous study in the emergency department setting found that the incidence of severe sepsis defined using traditional methods significantly increased from 1994 and 2009, while that for more explicitly-defined sepsis remained stable [24]. These data collectively suggest that the higher incidence rates of septic shock might be attributable to artifact of a change in coding practice, a growing awareness of septic shock, and possible financial incentives. Indeed, a recent literature has emphasized that the differences in mortality of septic shock caused by coding practice [25, 26]. These factors would lead to an increased use of diagnosis codes that classify hospitalizations as being related to sepsis [8, 9, 27]. Consequently, patients with a lower illness severity who previously were not identified as having the condition would be included in more recent years [2, 6, 7]. This influx of less severe cases would thereby lead to an apparent decrease in mortality trend.

To address these concerns, surveillance definitions that are simple, objective, clinically meaningful, and resistant to ascertainment bias are important [9]. We believe our restrictive definition (i.e., major sepsis-causing infections with the use of vasopressor therapy) meets these criteria and is thus a more specific method for the identification of patients with septic shock for the following reasons. First, focusing on the four major causes for sepsis eliminates the use of obscure infection-related diagnosis codes that fall under “suspected infection” [11]. Second, clinical consensus is that infected patients who require vasopressors have clinically-significant septic shock, and this case definition has been used in previous studies [3, 13]. Finally, a study period starting in 2005 provides consistency of our definitions over the study period given that codes for septic shock and vasopressor use were first introduced in 2003. Therefore, our findings are more likely to reflect the mortality trends of a like sepsis population over the entire study period compared to prior studies [1, 2, 6, 7].

The reasons for the observed lack of mortality improvement in the subgroup with vasopressor use are likely multifactorial. First, in contrast to the previous studies using *ICD-9-CM* codes linking infection to new organ dysfunction as way to identify severe sepsis and septic shock [2, 6, 7, 10], we focused on the sicker population. Patients with septic shock requiring vasopressors are critically ill, and the mortality exceeds 40 % in recent years [3, 11]. Second, although substantial investment of resources and advancement in knowledge have undoubtedly improved the day-to-day care of critically ill patients, management continues without a decisive therapy for septic shock; multiple large trials have failed to improve the case fatality of septic shock [13, 28–33]. It is also plausible that a gap in intensive care resources (e.g., number of intensive care physicians, number of intensive care beds) [34–38] across the nation might have contributed to the observed lack of improvement in the mortality. As septic shock is a common condition that requires a large proportion of healthcare resources, these documented disparities in intensive care resources may pose a roadblock to decreasing nationwide mortality. Moreover, although the implementation of guidelines and bundles is known to decrease mortality in the intensive care units [39, 40], the benefit of guidelines are hampered by the poor adoption in both resource-rich and resource-poor environments [41]. Indeed, a study of 165 sites documented that adherence to the entire management bundle recommended in the Surviving Sepsis Campaign is only 36 % [42].

Because septic shock is a relatively common public health problem, and any intensive care unit has accountability to provide best practice, our data, in conjunction with the current literature [9, 29, 30, 38, 39], underscore the need for continued efforts to improve systems of care —e.g., an enrichment of intensive care resource and standardization of management with implementation of the clinical guidelines. Sepsis presents a great opportunity for such quality improvements.

Our study has several potential limitations. First, the NIS does not provide granular information on clinical or physiologic measurements, other drugs administered, or Do-Not-Attempt-Resuscitation codes, as these clinical data are not captured in the *ICD-9-CM* codes. These factors might have confounded our inferences; however, we controlled for Elixhauser comorbidity measures as a surrogate for patient comorbidity in our analysis [43]. Second, the number of patients with septic shock in our study was smaller compared to other studies [1, 4, 7]. It is likely that vasopressor use was under-reported in our study, especially in the first years after adoption of the new procedure code for vasopressor use. Nevertheless, our objective was not to examine the incidence but to investigate the mortality trend in patients with septic shock who requires the use of vasopressors. And we found no significant change in mortality in this specific group of patients. Third, the lack of longitudinal follow-up data in the HCUP database precludes us from examining longer-term outcomes. However, the HCUP data are widely used to investigate the mortality trends [1, 10], thereby we used in-hospital mortality to maintain the consistency with the previous literature. Fourth, we did not examine the differences in mortality between patients with the primary diagnosis of infection and those with the primary diagnosis of “septic shock” because of the proportion of patients with the primary diagnosis of septic shock was less than 1 %. Nevertheless, a recent literature has investigated the differences by using similar HCUP datasets [25], and consistent with our observation. Finally, as our findings focused on the four major infections, caution is required when extrapolating our results to patients with septic shock caused by other infections and comparing to previous observation using the primary diagnosis of sepsis, severe sepsis, or septic shock [1, 10, 25, 44]. However, the four selected infections account for more than 80 % of septic shock; therefore, our data are of likely relevance to most patients with septic shock [45].

## Conclusions

In sum, using a nationally-representative sample, we found an approximate 10 % relative decrease in in-hospital mortality among all patients identified as septic shock from 2005 to 2011. However, in the subgroup with vasopressor use (i.e., major sepsis-causing infection plus the use of a vasopressor), we found no change in in-hospital mortality during the same period, thus challenging the conventional wisdom that mortality in this population has improved. The case fatality of septic shock remains unacceptably high over the study period. For researchers, our observations should motivate further investigation of barriers to the delivery of high-quality sepsis care and the development of novel therapeutic strategies. Because septic shock is an ongoing significant public health burden, policymakers will need to develop better surveillance systems and promote continued efforts to improve systems of care.

## Abbreviations

AHRQ, Agency for Healthcare Research and Quality’s; ICD-9-CM, International Classification of Diseases 9th Revision Clinical Modification; NIS, Nationwide Inpatient Sample; US, United States.

## Additional file

Additional file 1: Table S1.
*International Classification of Diseases, Ninth Revision, Clinical Modification* codes for major causes of sepsis. **Table S2.** Patient and hospital characteristics in the subgroup with vasopressor use, 2005-2011. **Table S3.** Patient and hospital characteristics in the subgroup without vasopressor use, 2005-2011. **Table S4.** Unadjusted mortality among patients hospitalized for septic shock in the US, 2005-2011. **Table S5.** Odds ratio of inhospital mortality in patients hospitalized for septic shock in the US, 2005-2011.
