# Supplementary material for: Mortality trends in U.S. adults with septic shock, 2005-2011: a serial cross-sectional analysis of nationally-representative data
Source: BMC Infect Dis. 2016 Jun 14;16:294. doi: 10.1186/s12879-016-1620-1 (PMC4908776; doi:10.1186/s12879-016-1620-1)
Supplement: Additional file 1: Table S1. — International Classification of Diseases, Ninth Revision, Clinical Modification codes for major causes of sepsis. Table S2. Patient and hospital characteristics in the subgroup with vasopressor use, 2005-2011. Table S3. Patient and hospital characteristics in the subgroup without vasopressor use, 2005-2011. Table S4. Unadjusted mortality among patients hospitalized for septic shock in the US, 2005-2011. Table S5. Odds ratio of inhospital mortality in patients hospitalized for septic shock in the US, 2005-2011. [file 12879_2016_1620_MOESM1_ESM.docx]

**Additional tables**

**Additional Table 1. *International Classification of Diseases, Ninth Revision, Clinical Modification* codes for Major Causes of Sepsis**

| Infection | *ICD-9-CM* codes |
| --- | --- |
| Pneumonia | 481, 482, 483, 485, 486 |
| Urinary tract infection | 590, 595.0, 595.2-4, 595.89, 595.9, 597, 598.00-01, 599.0 |
| Abdominal infection |  |
| Hepatobiliary infection | 572, 574-576 |
| Abdominal and rectal | 540-542, 543.9, 562.01, 562.03, 562.11, 562.13, 567, 569.5, 569.61, 569.71, 569.83 |
| Enteric infection | 008.45, 009 |
| Inflammatory disease of female pelvic organs | 614, 616 |
| Bacteraemia | 790.7 |

**Additional Table 2. Patient and Hospital Characteristics in the Subgroup With Vasopressor Use, 2005-2011.**

| Variables^*^ | 2005 | 2006 | 2007 | 2008 | 2009 | 2010 | 2011 | P_trend_ |
| --- | --- | --- | --- | --- | --- | --- | --- | --- |
| **Patient characteristics** |  |  |  |  |  |  |  |  |
| Unweighted sample, n | 484 | 523 | 545 | 518 | 559 | 645 | 607 |  |
| Weighted sample, n | 2392 | 2566 | 2664 | 2494 | 2789 | 3293 | 2910 |  |
| Age, y |  |  |  |  |  |  |  |  |
| 18-29 | 1 (1-2) | 1 (0-2) | 2 (1-3) | 2 (1-4) | 1 (1-3) | 1 (1-3) | 2 (1-3) | 0.39 |
| 30-39 | 3 (1-5) | 2 (1-4) | 3 (1-5) | 3 (2-5) | 2 (1-4) | 3 (2-5) | 3 (2-4) | 0.60 |
| 40-49 | 5 (3-8) | 9 (7-11) | 8 (6-11) | 5 (4-8) | 5 (3-7) | 7 (5-9) | 8 (5-10) | 0.91 |
| 50-59 | 12 (8-17) | 13 (11-17) | 14 (11-17) | 16 (13-21) | 15 (12-18) | 14 (11-18) | 16 (13-20) | 0.13 |
| 60-69 | 19 (16-21) | 18 (15-21) | 20 (16-24) | 20 (16-24) | 22 (19-25) | 22 (19-26) | 27 (23-31) | <0.001 |
| 70-79 | 28 (24-32) | 24 (21-28) | 26 (23-30) | 27 (23-31) | 26 (23-30) | 26 (23-30) | 22 (19-25) | 0.11 |
| 80-89 | 27 (22-32) | 26 (22-30) | 22 (18-27) | 22 (17-27) | 23 (19-28) | 22 (19-26) | 20 (17-24) | 0.02 |
| ≥90 | 6 (4-9) | 7 (5-10) | 5 (4-7) | 5 (3-7) | 6 (4-8) | 4 (2-6) | 3 (2-5) | 0.004 |
| Male sex | 49 (46-53) | 55 (50-59) | 48 (44-52) | 49 (45-53) | 50 (46-54) | 51 (48-55) | 51 (46-55) | 0.94 |
| Race/ethnicity |  |  |  |  |  |  |  |  |
| Non-Hispanic white | 72 (63-80) | 61 (50-71) | 60 (51-69) | 60 (50-70) | 77 (69-84) | 71 (65-77) | 66 (59-74) | 0.31 |
| Non-Hispanic black | 7 (4-12) | 8 (5-13) | 11 (6-19) | 7 (4-12) | 3 (2-5) | 12 (8-18) | 10 (8-14) | 0.25 |
| Hispanic | 4 (2-10) | 7 (4-11) | 6 (3-12) | 6 (3-10) | 5 (3-10) | 9 (5-15) | 9 (6-14) | 0.08 |
| Asian/native/other | 4 (2-6) | 6 (3-10) | 5 (4-10) | 3 (2-6) | 5 (3-8) | 4 (2-5) | 7 (4-10) | 0.48 |
| Unknown | 13 (7-23) | 19 (10-32) | 17 (10-28) | 23 (14-37) | 9 (4-18) | 4 (2-7) | 7 (2-16) | 0.008 |
| Primary health insurance |  |  |  |  |  |  |  |  |
| Medicare | 73 (69-78) | 69 (64-74) | 68 (63-73) | 66 (60-71) | 70 (65-74) | 66 (61-71) | 65 (60-70) | 0.04 |
| Medicaid | 7 (4-10) | 7 (5-11) | 6 (4-11) | 8 (6-11) | 8 (6-10) | 9 (7-12) | 11 (8-14) | 0.02 |
| Private | 18 (14-22) | 18 (15-22) | 21 (17-26) | 22 (17-28) | 18 (15-23) | 18 (15-22) | 17 (14-21) | 0.64 |
| Self-pay | 2 (1-4) | 2 (2-4) | 2 (1-3) | 2 (1-4) | 2 (1-4) | 4 (2-6) | 3 (2-6) | 0.08 |
| Other | 1 (0-2) | 3 (2-6) | 3 (2-5) | 2 (1-5) | 2 (1-4) | 3 (2-5) | 3 (2-5) | 0.11 |
| Estimated median household income |  |  |  |  |  |  |  |  |
| 0-25 percentile | 22 (15-33) | 27 (21-34) | 25 (19-34) | 24 (18-30) | 25 (20-30) | 33 (26-40) | 25 (20-32) | 0.31 |
| 26-50 percentile | 24 (17-34) | 25 (20-30) | 25 (20-31) | 23 (19-29) | 23 (19-29) | 20 (15-24) | 23 (19-28) | 0.24 |
| 51-75 percentile | 26 (19-33) | 24 (19-29) | 23 (19-28) | 25 (19-34) | 26 (21-33) | 24 (20-29) | 28 (23-33) | 0.45 |
| 76-100 percentile | 27 (17-42) | 24 (19-31) | 27 (20-35) | 28 (21-36) | 25 (20-32) | 23 (18-30) | 24 (19-31) | 0.57 |
| Selected comorbidities^†^ |  |  |  |  |  |  |  |  |
| Congestive heart failure | 39 (35-44) | 41 (36-45) | 38 (33-43) | 34 (28-41) | 37 (33-41) | 34 (30-38) | 34 (29-39) | 0.01 |
| Pulmonary circulation disorders | 3 (1-4) | 4 (2-5) | 9 (6-11) | 8 (5-11) | 11 (8-14) | 12 (10-15) | 12 (9-15) | <0.001 |
| Diabetes, uncomplicated | 16 (11-20) | 17 (14-20) | 17 (14-20) | 20 (17-23) | 21 (17-24) | 20 (16-23) | 24 (21-28) | <0.001 |
| Liver disease | 4 (1-6) | 4 (2-6) | 5 (3-8) | 5 (3-8) | 6 (4-9) | 6 (4-7) | 9 (6-11) | 0.003 |
| Renal failure | 15 (12-19) | 24 (21-27) | 24 (20-28) | 22 (18-27) | 25 (21-30) | 26 (22-30) | 26 (22-30) | 0.001 |
| Solid tumour without metastasis | 4 (2-5) | 4 (2-5) | 3 (1-4) | 5 (3-7) | 3 (2-5) | 4 (2-5) | 4 (3-6) | 0.50 |
| **Hospital characteristics** |  |  |  |  |  |  |  |  |
| Region |  |  |  |  |  |  |  |  |
| Northeast | 48 (29-67) | 35 (21-52) | 24 (14-38) | 18 (10-30) | 21 (12-36) | 23 (14-35) | 23 (14-35) | 0.05 |
| Midwest | 24 (11-43) | 19 (10-33) | 22 (13-36) | 26 (15-40) | 28 (18-41) | 26 (15-39) | 24 (15-37) | 0.59 |
| South | 16 (8-31) | 21 (13-33) | 31 (21-44) | 34 (22-50) | 30 (20-43) | 34 (23-48) | 27 (19-38) | 0.10 |
| West | 12 (6-23) | 26 (16-39) | 23 (14-33) | 22 (14-33) | 21 (13-31) | 18 (10-28) | 26 (17-37) | 0.49 |
| Location/teaching status |  |  |  |  |  |  |  |  |
| Rural | 2 (1-7) | 6 (2-14) | 9 (5-18) | 4 (2-11) | 10 (6-18) | 8 (4-17) | 8 (4-16) | 0.10 |
| Urban nonteaching | 45 (27-64) | 35 (23-49) | 33 (23-45) | 38 (26-52) | 37 (26-49) | 37 (26-50) | 43 (32-55) | 0.99 |
| Urban teaching | 53 (34-71) | 59 (45-72) | 57 (45-69) | 57 (44-70) | 53 (40-65) | 55 (42-67) | 49 (37-61) | 0.44 |
| Hospital control/ownership |  |  |  |  |  |  |  |  |
| Government | 3 (1-8) | 7 (3-15) | 5 (2-10) | 7 (3-15) | 8 (4-15) | 4 (2-8) | 7 (3-16) | 0.54 |
| Private, non-profit | 16 (7-32) | 18 (10-30) | 17 (10-27) | 17 (10-27) | 18 (10-29) | 17 (9-28) | 16 (10-24) | 0.86 |
| Private, invest-own | 1 (0-5) | 3 (1-8) | 5 (2-11) | 2 (1-6) | 7 (2-17) | 8 (3-16) | 4 (2-9) | 0.07 |
| Others | 80 (64-89) | 72 (59-83) | 73 (62-82) | 74 (62-83) | 68 (56-78) | 72 (60-82) | 73 (62-81) | 0.35 |

Data are expressed as % (95% CI) unless otherwise indicated.

*Percentages may not equal 100 due to rounding.

**Additional Table 3. Patient and Hospital Characteristics in the Subgroup Without Vasopressor Use, 2005-2011.**

| Variables^*^ | 2005 | 2006 | 2007 | 2008 | 2009 | 2010 | 2011 | P_trend_ |
| --- | --- | --- | --- | --- | --- | --- | --- | --- |
| **Patient characteristics** |  |  |  |  |  |  |  |  |
| Unweighted sample, n | 2343 | 2260 | 2451 | 2863 | 2879 | 2797 | 2786 |  |
| Weighted sample, n | 11554 | 11062 | 12198 | 14069 | 14450 | 13999 | 13373 |  |
| Age, y |  |  |  |  |  |  |  |  |
| 18-29 | 2 (1-3) | 2 (1-2) | 2 (2-3) | 2 (2-3) | 2 (2-3) | 2 (2-3) | 2 (1-3) | 0.62 |
| 30-39 | 3 (3-4) | 4 (3-4) | 3 (3-4) | 3 (2-3) | 3 (3-4) | 3 (2-4) | 3 (2-3) | 0.14 |
| 40-49 | 8 (7-9) | 8 (7-9) | 9 (8-11) | 8 (7-9) | 7 (6-8) | 8 (7-9) | 7 (6-8) | 0.19 |
| 50-59 | 16 (15-18) | 17 (15-19) | 15 (13-16) | 15 (14-17) | 17 (16-18) | 15 (14-16) | 16 (15-18) | 0.90 |
| 60-69 | 19 (17-21) | 20 (18-21) | 20 (18-22) | 21 (20-23) | 21 (20-23) | 22 (20-24) | 23 (22-25) | <0.001 |
| 70-79 | 25 (23-27) | 24 (23-26) | 24 (23-26) | 23 (22-25) | 23 (22-25) | 24 (22-26) | 23 (21-24) | 0.06 |
| 80-89 | 23 (21-25) | 22 (20-24) | 21 (19-23) | 22 (20-23) | 21 (20-23) | 21 (19-23) | 21 (19-23) | 0.19 |
| ≥90 | 4 (3-5) | 5 (4-6) | 5 (4-6) | 5 (4-6) | 5 (4-6) | 5 (5-6) | 5 (4-6) | 0.43 |
| Male sex | 52 (49-54) | 51 (48-53) | 51 (49-53) | 50 (48-52) | 50 (48-52) | 50 (48-52) | 49 (47-51) | 0.07 |
| Race/ethnicity |  |  |  |  |  |  |  |  |
| Non-Hispanic white | 59 (55-63) | 52 (48-57) | 53 (49-57) | 57 (53-61) | 60 (56-65) | 62 (58-66) | 64 (60-68) | <0.001 |
| Non-Hispanic black | 7 (6-9) | 10 (8-12) | 9 (7-11) | 9 (7-11) | 9 (8-11) | 11 (10-14) | 11 (10-13) | 0.002 |
| Hispanic | 8 (6-10) | 11 (8-14) | 9 (7-12) | 9 (7-11) | 10 (8-12) | 10 (8-13) | 11 (8-13) | 0.20 |
| Asian/native/other | 5 (4-6) | 5 (4-6) | 6 (5-7) | 6 (5-8) | 6 (5-8) | 6 (5-8) | 6 (4-7) | 0.11 |
| Unknown | 21 (17-26) | 22 (18-27) | 23 (19-27) | 19 (15-24) | 14 (10-20) | 10 (7-14) | 8 (6-12) | <0.001 |
| Primary health insurance |  |  |  |  |  |  |  |  |
| Medicare | 64 (62-67) | 64 (62-67) | 64 (61-66) | 64 (61-66) | 64 (62-66) | 64 (61-66) | 64 (62-66) | 0.86 |
| Medicaid | 11 (9-12) | 11 (9-14) | 10 (8-12) | 11 (10-13) | 10 (9-11) | 11 (10-13) | 11 (9-12) | 0.95 |
| Private | 19 (17-21) | 18 (16-20) | 20 (18-22) | 20 (18-21) | 20 (18-22) | 18 (16-20) | 19 (18-21) | 0.84 |
| Self-pay | 4 (3-5) | 4 (3-5) | 4 (3-5) | 4 (3-4) | 4 (3-5) | 4 (3-5) | 3 (2-4) | 0.18 |
| Other | 2 (2-3) | 3 (2-4) | 2 (2-3) | 2 (1-3) | 2 (2-3) | 3 (2-3) | 3 (2-4) | 0.33 |
| Estimated median household income |  |  |  |  |  |  |  |  |
| 0-25 percentile | 27 (24-31) | 28 (24-31) | 30 (26-34) | 29 (26-33) | 29 (26-32) | 29 (26-32) | 29 (26-32) | 0.37 |
| 26-50 percentile | 27 (24-30) | 25 (22-28) | 25 (23-28) | 26 (23-29) | 25 (23-28) | 26 (24-28) | 24 (22-27) | 0.49 |
| 51-75 percentile | 23 (21-26) | 24 (22-27) | 24 (21-26) | 22 (19-24) | 23 (21-25) | 24 (22-26) | 27 (25-29) | 0.13 |
| 76-100 percentile | 23 (19-26) | 23 (19-27) | 21 (18-24) | 23 (20-27) | 23 (20-27) | 22 (19-25) | 19 (17-22) | 0.25 |
| Selected comorbidities^†^ |  |  |  |  |  |  |  |  |
| Congestive heart failure | 29 (26-31) | 29 (27-31) | 29 (27-31) | 25 (23-27) | 27 (25-29) | 26 (24-28) | 30 (28-32) | 0.44 |
| Pulmonary circulation disorders | 2 (1-3) | 2 (2-3) | 4 (3-5) | 5 (4-6) | 6 (5-7) | 6 (5-7) | 7 (6-8) | <0.001 |
| Diabetes, uncomplicated | 15 (13-17) | 16 (15-18) | 18 (16-20) | 18 (17-20) | 19 (17-21) | 17 (16-19) | 20 (18-22) | <0.001 |
| Liver disease | 6 (5-7) | 5 (4-6) | 5 (4-6) | 5 (4-6) | 6 (5-7) | 6 (5-7) | 7 (5-8) | 0.03 |
| Renal failure | 14 (12-16) | 19 (18-21) | 21 (19-23) | 21 (19-23) | 22 (20-24) | 21 (19-22) | 24 (22-25) | <0.001 |
| Solid tumour without metastasis | 5 (4-5) | 3 (3-4) | 5 (4-6) | 4 (3-5) | 5 (4-6) | 5 (4-6) | 5 (5-6) | 0.007 |
| **Hospital characteristics** |  |  |  |  |  |  |  |  |
| Region |  |  |  |  |  |  |  |  |
| Northeast | 26 (21-31) | 24 (19-30) | 24 (19-29) | 22 (18-28) | 20 (16-25) | 22 (17-27) | 19 (14-26) | 0.09 |
| Midwest | 21 (17-25) | 21 (17-26) | 21 (17-26) | 24 (20-30) | 25 (20-30) | 22 (18-27) | 23 (19-29) | 0.40 |
| South | 33 (28-39) | 33 (28-38) | 31 (26-37) | 32 (27-37) | 32 (27-38) | 37 (32-43) | 40 (34-46) | 0.05 |
| West | 20 (16-26) | 22 (17-27) | 24 (19-30) | 21 (17-27) | 23 (19-28) | 19 (15-24) | 18 (14-22) | 0.26 |
| Location/teaching status |  |  |  |  |  |  |  |  |
| Rural | 13 (11-16) | 9 (7-11) | 11 (8-13) | 9 (7-11) | 9 (7-11) | 10 (8-13) | 10 (8-12) | 0.13 |
| Urban nonteaching | 48 (42-54) | 43 (38-49) | 45 (39-50) | 43 (38-49) | 46 (41-52) | 44 (39-50) | 43 (37-48) | 0.36 |
| Urban teaching | 39 (33-45) | 48 (42-53) | 45 (39-51) | 48 (42-55) | 45 (39-51) | 46 (40-52) | 48 (42-55) | 0.25 |
| Hospital control/ownership |  |  |  |  |  |  |  |  |
| Government | 6 (4-9) | 5 (4-7) | 5 (4-7) | 5 (4-7) | 5 (4-7) | 5 (4-7) | 5 (3-8) | 0.83 |
| Private, non-profit | 23 (18-28) | 18 (15-23) | 16 (13-20) | 18 (14-22) | 18 (14-22) | 18 (14-22) | 17 (13-21) | 0.12 |
| Private, invest-own | 7 (6-9) | 10 (7-13) | 12 (9-16) | 11 (8-14) | 12 (9-15) | 11 (8-15) | 13 (10-17) | 0.03 |
| Others | 64 (59-69) | 67 (62-72) | 67 (62-72) | 67 (62-72) | 65 (60-70) | 66 (61-71) | 65 (60-70) | 0.75 |

Data are expressed as % (95% CI) unless otherwise indicated.

*Percentages may not equal 100 due to rounding.

**Additional Table 4. Unadjusted Mortality Among Patients Hospitalized for Septic Shock in the US, 2005-2011.**

| Mortality | 2005 | 2006 | 2007 | 2008 | 2009 | 2010 | 2011 | P_trend_ |
| --- | --- | --- | --- | --- | --- | --- | --- | --- |
| Overall | 46 (46-46) | 46 (46-46) | 43 (43-43) | 45 (44-45) | 44 (44-45) | 43 (43-43) | 42 (42-42) | 0.003 |
| Subgroup with vasopressor use | 42 (42-42) | 41 (40-41) | 39 (39-39) | 42 (42-42) | 42 (42-42) | 39 (39-39) | 40 (40-40) | 0.57 |
| Subgroup without vasopressor use | 47 (47-47) | 47 (47-47) | 45 (45-45) | 46 (46-46) | 46 (46-46) | 44 (44-44) | 43 (43-43) | 0.002 |

Data are expressed as % (95% CI).

**Additional Table 5. Odds Ratio of Inhospital Mortality in Patients Hospitalized for Septic Shock in the US, 2005-2011.**

|  | 2005 | 2006 | 2007 | 2008 | 2009 | 2010 | 2011 | Per year |
| --- | --- | --- | --- | --- | --- | --- | --- | --- |
| Unadjusted odds ratio (95% CI) | |  |  |  |  |  |  |  |
| Overall | 1 [reference] | 0.99 (0.87-1.12) | 0.90 (0.81-1.01) | 0.95 (0.84-1.07) | 0.95 (0.84-1.07) | 0.88 (0.78-0.99) | 0.85 (0.75-0.95) | 0.98 (0.96-0.99) |
| Subgroup with vasopressor use | 1 [reference] | 0.92 (0.69-1.23) | 0.88 (0.67-1.16) | 0.97 (0.74-1.26) | 0.99 (0.76-1.30) | 0.88 (0.67-1.16) | 0.89 (0.67-1.18) | 0.99 (0.95-1.03) |
| Subgroup without vasopressor use | 1 [reference] | 1.01 (0.88-1.15) | 0.91 (0.80-1.03) | 0.94 (0.82-1.08) | 0.94 (0.82-1.07) | 0.88 (0.77-1.00) | 0.84 (0.74-0.95) | 0.97 (0.96-0.99) |
| Adjusted odds ratio (95% CI) | |  |  |  |  |  |  |  |
| Overall | 1 [reference] | 1.01 (0.89-1.15) | 0.90 (0.80-1.00) | 1.01 (0.89-1.14) | 0.99 (0.88-1.12) | 0.87 (0.77-0.99) | 0.87 (0.78-0.99) | 0.98 (0.96-1.00) |
| Subgroup with vasopressor use | 1 [reference] | 1.08 (0.79-1.48) | 0.94 (0.70-1.25) | 1.11 (0.84-1.48) | 1.19 (0.89-1.60) | 1.00 (0.74-1.35) | 1.08 (0.81-1.44) | 1.01 (0.97-1.05) |
| Subgroup without vasopressor use | 1 [reference] | 1.01 (0.88-1.16) | 0.89 (0.79-1.00) | 0.98 (0.86-1.12) | 0.96 (0.84-1.10) | 0.85 (0.75-0.98) | 0.84 (0.73-0.95) | 0.97 (0.95-0.99) |
